# Actinomyces as a rare cause of appendicitis: a case report

**DOI:** 10.1093/jscr/rjae195

**Published:** 2024-04-01

**Authors:** Yijun Gao, Shaurya Jhamb, Raymond Hayler, Chloe Trickett, Allan Kwok

**Affiliations:** Department of Surgery, The Sutherland Hospital, The Kingsway, Caringbah 2229, NSW, Australia; Department of Surgery, The Sutherland Hospital, The Kingsway, Caringbah 2229, NSW, Australia; Department of Surgery, St George Public Hospital, Gray Street, Kogarah 2217, NSW, Australia; Department of Surgery, The Sutherland Hospital, The Kingsway, Caringbah 2229, NSW, Australia; Department of Surgery, The Sutherland Hospital, The Kingsway, Caringbah 2229, NSW, Australia

## Abstract

Actinomycosis remains a rare and often underdiagnosed cause of appendicitis with only 10% of cases diagnosed prior to surgery. It is an important cause to consider particularly in the setting of an indolent infection with nonspecific symptoms. We present a 22 years old male who presented with 3 weeks history of lower abdominal pain who underwent laboratory investigations and imaging studies suggestive of acute appendicitis. He underwent an emergency laparoscopic caecectomy with histopathology of the specimen suggestive of actinomycosis. He recovered well postoperatively and was discharged home with a prolonged course of oral penicillins. Preoperative diagnosis of actinomycosis is uncommon and accounts for ~10% of cases. Definitive diagnosis is usually through histopathology or tissue/fluid culture. Treatment usually involves a combination of surgical resection and antibiotic therapy with a success rate of >90%.

## Introduction

*Actinomyces* is a gram-positive anaerobic bacteria that are normal commensals of the oral cavity, the upper respiratory and gastrointestinal tracts and the female genital tract [1]. Actinomycosis is an indolent, slowly progressive infection caused by the bacteria and often presents with nonspecific symptoms which can mimic inflammatory bowel disease or diverticulitis. The appendix and ileocaecal region are most commonly involved however cases of hepatic, retroperitoneal, and adrenal actinomycosis have also been reported [2]. Actinomycosis is rare with an incidence between 1 in 300 000 and 1 in 1 000 000 and occurs when the normal mucosal barrier is breached. It is estimated to represent ~0.02% of causes of acute appendicitis [3]. Predisposing factors include recent abdominal surgery, bowel perforation, neoplasia, poor oral hygiene, and intrauterine contraceptive devices [2].

Acute appendicitis is the most common indication in the world for emergency abdominal surgery and carries a lifetime incidence of 10%. The pathophysiology of appendicitis involves luminal obstruction with common causes including lymphoid hyperplasia and faecal stasis with faecoliths. Carcinoid tumors, eosinophilic appendicitis, granulomas and actinomycosis are rarer causes.

Here we present a case of subacute appendicitis secondary to *Actinomyces* diagnosed after surgery, as well as a literature review of this rare entity.

## Case

**Figure 1:**
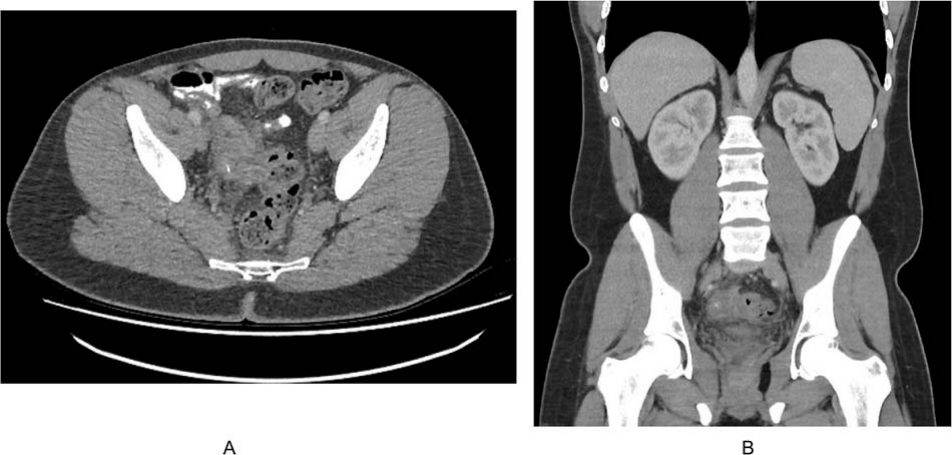
(A). Axial slice of a CT abdomen/pelvis in the portovenous phase demonstrating an inflammatory mass in the pelvis associated with the right pelvic side wall, with a calcific focus. (B). Coronal slice of the CT demonstrating the relation of the inflammatory mass within the pelvis.

A 22 years old male with no prior medical or surgical history presented to the emergency department with 3 weeks history of lower abdominal pain and rectal discomfort. The pain was noted to be more suprapubic rather than the right iliac fossa but his abdomen was soft with no features of peritonism. He denied fevers, nausea or vomiting, or changes to bowel habits. There was no family history of bowel cancers or inflammatory bowel disease. His observations on admission were unremarkable. Pathology testing revealed a white cell count of 8.30 x 10^9^/L and a c-reactive protein of 22 mg/L. An initial ultrasound of the abdomen visualized a non-compressible tubular structure measuring 9 mm in the right iliac fossa with 4.4 ml of surrounding free fluid and prominent lymph nodes. Given the atypical history for appendicitis, a computed tomography of the abdomen and pelvis was performed which demonstrated significant inflammatory change close to the terminal ileum extending to the right pelvic side wall with a calcific focus possibly representing an appendicolith (Fig. 1). The actual appendix was not seen as a distinct entity and the actual terminal ileum itself was not thickened.

**Figure 2:**
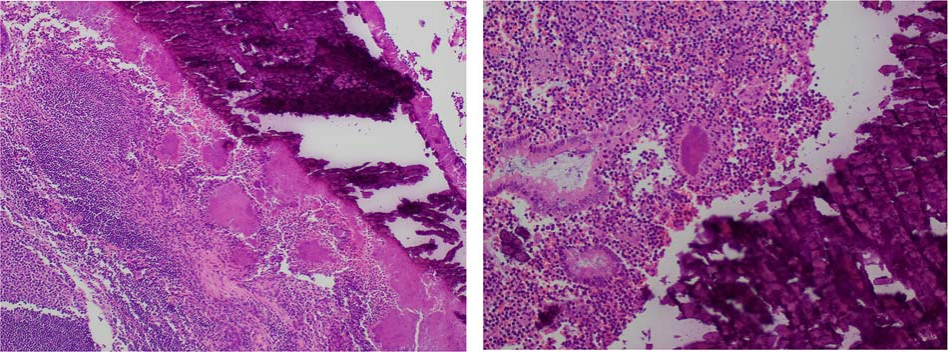
Microscopy slides demonstrating a luminal faecolith surrounded by Actinomyces.

He underwent an emergency laparoscopic stapled caecectomy. Intraoperatively, the appendix was firmly adherent to the terminal ileum and right pelvic side wall, forming a dense fibrotic mass. This was bluntly dissected and given the appearance of an oedematous appendiceal base, the decision was made for a stapled caecectomy. Postoperatively, the patient recovered well and was discharged 3 days later. Interestingly, the histopathology of the specimen was reported as acute appendicitis with acute on chronic inflammation in the appendiceal mucosa and a luminal faecolith surrounded by actinomyces-like organisms (Fig. 2). There was no mention of sulfur granules on the pathology report. Advice was sought from the infectious diseases specialists and the patient was placed on a course of oral penicillins. The patient has ongoing follow up to ensure no recurrence of disease.

## Discussion

Actinomycosis occurs commonly in three distinct forms—cervicofacial presentation in 50% of cases, abdominopelvic in 20%, and intrathoracic in 15% [3]. *Actinomyces israelii* is the most common aetiologic organism of actinomycosis. In abdominal actinomycosis, the appendix is perforated in ~75% of cases as *Actinomyces* requires injury to the mucosa to penetrate and cause disease and hence an inflamed but intact appendix is a rare find [4]. In our case, the appendix remained intact without histopathological features of perforation.

The clinical presentation is often indolent with non-specific symptoms and <10% of cases of actinomycosis is discovered prior to surgery. Laboratory investigations offer little value in diagnosis and imaging is useful in determining the location and extent of disease but remain nonspecific. On CT imaging, actinomycosis often resembles a solid mass with prominent contrast enhancement [5]. Small rim-enhancing lesions are sometimes found in the solid portion of the mass which are thought to represent small abscesses. A definitive diagnosis is usually obtained via visualization of characteristic sulfur granules or culture of *Actinomyces* obtained from a collection or surgical specimen on selective agar medium under anaerobic conditions for a minimum of 7 days [2]. Acute *Actinomyces* infection of the appendix can be differentiated from ileocaecal actinomycosis by *Actinomyces* granules that are detected in the appendiceal lumen [6].

The mainstay of treatment is with prolonged course penicillin via either an oral or intravenous route depending on severity of infection. Currently no guideline exists on the duration of treatment and that determination relies on clinical and radiological response to treatment [2]. Preceding surgical resection may allow for a shorter antibiotic duration [7]. A combination of antibiotics and surgical resection results in >90% success rate in treatment. Insufficient treatment may result in long-lasting disease, frequently seen as local dissemination or metastatic abscesses [6].

## Conclusion

Actinomycosis is a rare cause for acute appendicitis and should be consider when a patient presents with atypical symptoms but radiological features of appendicitis. Surgery remains a valuable tool in definitive diagnosis as well as an adjunct in therapy.
